# Interactions and Dissociation Constants of Galactomannan Rendered Cellulose Films with Concavalin A by SPR Spectroscopy

**DOI:** 10.3390/polym12123040

**Published:** 2020-12-18

**Authors:** Pilar Vilaró, Carina Sampl, Gundula Teichert, Werner Schlemmer, Mathias Hobisch, Michael Weissl, Luis Panizzolo, Fernando Ferreira, Stefan Spirk

**Affiliations:** 1 Sede Tacuarembó, Espacio de Ciencia y Tecnología Química, Universidad de la República, CENUR Nores-te. Ruta 5 Km 386, Tacuarembó 45000, Uruguay; pilar.vilaro@cut.edu.uy (P.V.); ff@fq.edu.uy (F.F.); 2 Institute of Bioproducts and Paper Technology, Graz University of Technology, Inffeldgasse 23, 8010 Graz, Austria; carina.sampl@tugraz.at (C.S.); gundual.teichert@student.tugraz.at (G.T.); werner.schlemmer@tugraz.at (W.S.); mathias.hobisch@tugraz.at (M.H.); michael.weissl@anton-paar.com (M.W.); 3 Departamento de Ciencia y Tecnología de Alimentos, Facultad de Química, Universidad de la República, Avenida General Flores 2124, Montevideo 11800, Uruguay; apanizzo@fq.edu.uy

**Keywords:** galactomannan, cellulose thin films, lectins

## Abstract

Interactions of biomolecules at interfaces are important for a variety of physiological processes. Among these, interactions of lectins with monosaccharides have been investigated extensively in the past, while polysaccharide-lectin interactions have scarcely been investigated. Here, we explore the adsorption of galactomannans (GM) extracted from *Prosopis affinis* on cellulose thin films determined by a combination of multi-parameter surface plasmon resonance spectroscopy (MP-SPR) and atomic force microscopy (AFM). The galactomannan adsorbs spontaneously on the cellulose surfaces forming monolayer type coverage (0.60 ± 0.20 mg·m^−2^). The interaction of a lectin, Concavalin A (ConA), with these GM rendered cellulose surfaces using MP-SPR has been investigated and the dissociation constant K_D_ (2.1 ± 0.8 × 10^−8^ M) was determined in a range from 3.4 to 27.3 nM. The experiments revealed that the galactose side chains as well as the mannose reducing end of the GM are weakly interacting with the active sites of the lectins, whereas these interactions are potentially amplified by hydrophobic effects between the non-ionic GM and the lectins, thereby leading to an irreversible adsorption.

## 1. Introduction

Hemicelluloses are important constituents of plant cell walls and feature rich structural diversity. For instance, xylans are prominent examples in soft and hard wood [1,2,3,4,5] while other hemicelluloses such as galactomannans (GM) mainly occur in the endosperm [3,5,6]. The structure of GM is composed of a *β*-1,4-D-mannopyranosyl backbone with *α*-D-galactopyranosyl residues attached to C6 of the mannosyl units [7]. The main structural motifs of GMs are classified according to the ratio between the galactose and the mannose units, usually referred to as M/G. The M/G arises from its fine distribution pattern and thereby influences its supramolecular properties [7,8,9]. Commercially available GM such as locust bean gum and guar gum feature M/G ratios of 3.5:1 and 1.5:1, respectively [10,11,12]. The galactose side chains impede interactions between mannose backbones and therefore significantly reduce the degree of entanglement between the macromolecules [7,13,14]. Therefore, the galactose content must be rather low to maximize mannose type cooperative interactions leading to higher viscosities, better gelling behavior and good film formation properties [15]. Typically, mannose type interactions are dominant if there are blocks of six mannose units present in the main chain. This reduces the tendency of GM to form tightly-packed aggregates playing a critical role for interactions with other polysaccharides [7,10]. Further, the degree of polymerization (DP) points a similar effect on the properties of the GMs; a usual range for GM is between 1000 and 1500 mannose units [9]. With increasing DP, the viscosity of a GM solution increases and films are exhibiting favorable characteristics in terms of mechanical properties compared to those obtained from GMs with a lower DP [9]. However, with a high galactose content and a very high DP, GM become less soluble, impeding the processing as emulsifiers or in film applications [16]. Some galactomannans play an important economic role on a regional level as they are enriched in the seeds of particular trees. *P. affinis*, for instance, is a tree species endemic in parts of South America, covering Uruguay, eastern Argentina and southern Brazil [17,18]. A single tree of this species can yield up to 100 kg of seeds, with 5 kg being the average for trees in plantation. It is estimated that in Uruguay there are approximately half a million naturally occurring *P. affinis* trees spread throughout the 852,000 ha of native woodland [19].

An interesting subject is how GM interact with proteins and here particularly with lectins. Lectins are interesting compounds, as they feature specific carbohydrate-protein interactions which are involved in many pathologic processes. However, their interaction potential at interfaces is only little explored. Valenga and coworkers for instance showed that monolayers of GM (from *Leucaena leucocephala* seeds) deposited on amino-terminated surfaces can be selectively modified with a lectin, Concavalin A (ConA), to serve as a support for the detection of dengue fever virus particles [20]. They used ex-situ methods to display the binding of the protein, such as atomic force microscopy and ellipsometry. Binding constants of ConA with the GM thin film have not been reported as the target was to find a platform for screening Dengue virus interaction. In the few other studies, ConA was immobilized on the surface and the binding constants with different types of mannans have been determined using QCM-D [21], a gravimetric method, that needs to adjust the interaction capacity for dissipation caused by swelling of the layers.

The importance of GM-protein interaction on one hand and the economic relevance of GM from *P. affinis* inspired us to have a closer look on the interaction behavior of GM at biopolymeric interfaces. We apply multi-parameter surface plasmon resonance spectroscopy (MP-SPR), an optical technique measuring only dry mass to better understand GM-cellulose and GM-lectin interactions at biopolymer interfaces. It further allows for monitoring real time interactions at interfaces, the approach comprises following steps: preparation of cellulose thin films resistant toward non-specific protein adsorption [22,23] (i), adsorption of GM onto the films (ii), and interaction of Concavaline A (ConA) with the GM rendered cellulose and pure cellulose films (iii). 

The novelty of our approach comes from three main points: (1) the GM from *P. affinis* has not been explored in any study concerning lectin interactions, (2) the method to determine the binding constants between ConA and the GM employs an optical technique (SPR spectroscopy) and (3) the support for the GM (cellulose) to explore the interaction with lectins has not been researched so far. The choice of this setup provides potential benefits as the support (cellulose) has low nonspecific protein affinity, SPR spectroscopy is a fast technique assess interactions at surfaces in real time and the immobilization of the GM on the surface allows for screening a large variety of different lectins to create a library of interaction potentials. Here, we show the proof of concept at the example of ConA as model lectin.

## 2. Materials and Methods

### 2.1. Materials

Purified galactomannans extracted from *P. affinis* at room temperature (GMP_1/RT_) with M/G at a ratio of 1.6, an average molar mass of 1.6 × 10^6^ g∙mol^−1^ (determined by SEC) [24], and a PDI of 0.3 (determined by DLS) were used. Trimethylsilyl cellulose (TMSC, Avicel, M_w_ = 185,000 g·mol^−1^, M_n_ = 30,400 g·mol^−1^, PDI = 6.1 determined by GPC in chloroform) with a DS_Si_ value of 2.8 was purchased from TITK (Rudolstadt, Germany). Chloroform (CHCl_3_, 99.3%), disodium phosphate heptahydrate (Na_2_HPO_4_ 7 H_2_O), sodium dihydrogen phosphate monohydrate (NaH_2_PO_4_·H_2_O), hydrochloric acid (HCl, 37%), sodium chloride (NaCl, Ph.Eur.), sodium hydroxide (NaOH, 99%), *Concanavalin A* (Type IV, lyophilized powder, 110 kDa) were purchased from Sigma Aldrich and used as received. Silicon wafers cut into 1.5 × 1.5 cm^2^ slides were used. SPR gold sensor slides (CEN102AU) were purchased from Cenibra, Germany. Milli-Q water (resistivity = 18.2 Ω^−1^·cm^−1^) from a Millipore water purification system (Millipore, St. Louis, MI, USA) was used for buffer and protein solution preparations.

### 2.2. Preparation of Solutions

For all experiments, a 10 mM phosphate buffer prepared from Milli-Q water exhibiting an ionic strength of 100 mM sodium chloride (adjusted with 0.1 M hydrochloric acid or 0.1 M NaOH) was used. ConA was dissolved in the buffer at a concentration of 0.1 mg·mL^−1^. The GM was dissolved in Milli-Q water by stirring for 12 h, at concentrations of 1 mg·mL^−1^ (for SPR adsorption) and 2 mg·mL^−1^ (for the spin coating process). 

### 2.3. Substrate Cleaning and Film Preparation

The SPR gold sensor slides and silicon wafer substrates were immersed in a “piranha” solution containing H_2_O_2_ (30 wt.%)/H_2_SO_4_ in a proportion of 1:3 *v*/*v* and 1:2 respectively for 10 min, subsequently extensively rinsed with Milli-Q water and blow dried in a stream of N_2_ gas as reported elsewhere [25]. For film preparation, 100 µL of TMSC solution (0.75 wt.% in chloroform) were deposited on the substrate and then rotated for 60 s at a spinning speed of 4000 rpm and an acceleration of 2500 rpm·s^−1^. TMSC was converted to cellulose by treatment with hydrochloric acid (HCl) vapor. For this purpose, the TMSC films were placed in a petri dish of 5 cm in diameter containing 3 mL of 10 wt.% HCl solution. After 12 min exposure to the HCl vapors in the covered dish, the regeneration was verified by water contact angle and ATR-IR measurements (Section 2.4) as reported elsewhere [25,26,27,28]. The film thickness of the films was 55 nm after regeneration.

### 2.4. Infrared Spectroscopy

IR spectra were attained by an Alpha FT-IR spectrometer (Bruker, Billerica, MA, USA) using an attenuated total reflection (ATR) attachment. Spectra were obtained in a scan range between 4000 to 400 cm^−1^ with 48 scans and a resolution of 4 cm^−1^. The data was analyzed with OPUS 4.0 software.

### 2.5. Profilometry

Film thicknesses were acquired with a DETAK 150 Stylus Profiler from Veeco. The scan length was set to 1000 µm over a duration of 3 seconds. Measurements were performed with a force of 3 mg, a resolution of 0.333 µm per sample and a measurement range of 6.5 µm. A diamond stylus with a radius of 12.5 µm was used. Samples were measured after scratching the film deposited on a silicon wafer. The resulting profile was used to calculate the thickness of the different films. All measurements were performed three times. 

### 2.6. Atomic Force Microscopy—AFM

Surface morphology and roughness of the films were obtained in tapping mode in ambient atmosphere at room temperature by a Veeco Multimode Quadrax MM scanning probe microscope (Bruker; Billerica, MA, USA) and by a Tosca TM 400 atomic force microscope (Anton Paar, Graz, Austria) using Si-cantilevers (NCH-VS1-W from NanoWorld AG, Neuchatel, Switzerland) with a resonance frequency of 320 kHz respectively 285 kHz and a force constant of 42 N·m^−1^. Root mean square (RMS) roughness calculation and image processing was performed with the Nanoscope software (V7.30r1sr3, Veeco, Plainview, NY, USA) and Gwyddion 2.53 (28.02.2019). 

### 2.7. Multi-Parameter Sruface Plasmon Resonance Spectroscopy: MP-SPR

MP-SPR spectroscopy was performed with a MP-SPR Navi^TM^ 210A Vasa instrument (from Bionavis Ltd., Tampere, Finland) equipped with two different lasers (670 and 785 nm, respectively) in both measurement channels, using gold coated glass slides as substrate (gold layer 50 nm, chromium adhesion layer 5 nm). All measurements were performed using a full angular scan (39–78°, scan speed: 8°·s^−1^). 

Gold sensor slides coated with a cellulose thin film were mounted in the SPR, equilibrated in Milli-Q water and afterwards in a 10 mM PBS (phosphate buffered saline) buffer with an ionic strength of 100 mM NaCl at pH 7.4. After equilibration, a solution of the GM (1.0 mg·mL^−1^) was injected into the flow cell for a period of at least 10 min and subsequently rinsed with corresponding buffer. Afterwards, a protein solution (1.0 mg·mL^−1^) was injected and allowed to adsorb for at least 10 min to the GM deposited on the cellulose film. The same results in GM deposition can be obtained when a solution of GM dissolved in MQ-water (1 mg·mL^−1^ GM, filtered) was dropped onto the regenerated cellulose surface, allowed to adsorb for a period of 10 min and then rinsed carefully with MQ-water. Regardless of the deposition method, the cellulose-galactomannan thin films were mounted in the SPR and equilibrated in a PBS buffer. After equilibration a solution containing ConA at different concentrations was allowed to adsorb for 10 min. The flow rate for all experiments was set to 20 µL·min^−1^. After the adsorption experiment the thin films were rinsed with buffer until the signal was completely stabilized. For the calculation of the amount of adsorbed galactomannan and protein, the shift of the SPR angle (ΔSPR) was used for each adsorption experiment. After protein adsorption, all samples were rinsed with Milli-Q water and dried in a stream of nitrogen. All experiments with ConA were performed in three parallels and GM adsorption on cellulose was done in 11 repetitions. 

Protein adsorption was quantified according to Equation (1), which considers the dependence of the angular response of the surface plasmon resonance in dependence of the refractive index increment (*dn*/*dc*) of the adsorbing layer.

(1) $$\Gamma =\;\frac{\Delta \Theta \;\times k\;\times \;d_{p}}{dn/dc}$$

For thin layers (<100 nm), *k* × *d_p_* can be considered constant and can be obtained by calibration of the instrument by determination of the peak wavelength of the resonance dip ld. For the MP-SPR NaviTM 210A Vasa used in this study, *k* × *d_p_* values are approximately 1.09 × 10^−7^ cm/° (at 670 nm) and 1.9 × 10^–7^ cm/° (at 785 nm) in aqueous systems. For ConA, *dn*/*dc* in water-based buffer systems was reported 0.187 cm^3^·g^−1^ which was used to calculate the amount of adsorbed masses. For GM, the *dn*/*dc* value of 0.145 cm^3^·g^–1^ was used for the calculation of adsorbed mass [29]. 

## 3. Results and Discussion

The cellulose substrates for the SPR studies were obtained by spin-coating an acid labile cellulose derivative, trimethylsilyl cellulose (TMSC) onto SPR gold sensor slides. After deposition of a TMSC film, the conversion to cellulose can be easily accomplished by exposure to HCl vapors as reported elsewhere [25,26,30,31]. This conversion accomplished by splitting the trimethylsilyl-groups, leads to a densification of the film, resulting in a reduction in film thickness by approximately 50% [26]. The reason for this reduction is a structural reorganization that occurs after the cleavage of the silyl groups due to the formation of inter- and intramolecular hydrogen bonds in cellulose [32]. The conversion is also associated with a change in the static water contact angles which changes from hydrophobic (95–100°) to hydrophilic (24–35°) [26,28,33,34]. The determination of contact angles with other liquids than water revealed disperse contributions to surface free energies, stemming from van der Waals interactions and London dispersion forces [35]. These films are particularly interesting as they provide a mostly amorphous matrix while featuring a smooth surface allowing for excluding roughness and morphology effects [36]. As the surfaces solely consist of cellulose, they represent an ideal model system to screen for interactions with polysaccharides and proteins [37,38].

**Figure 1:**
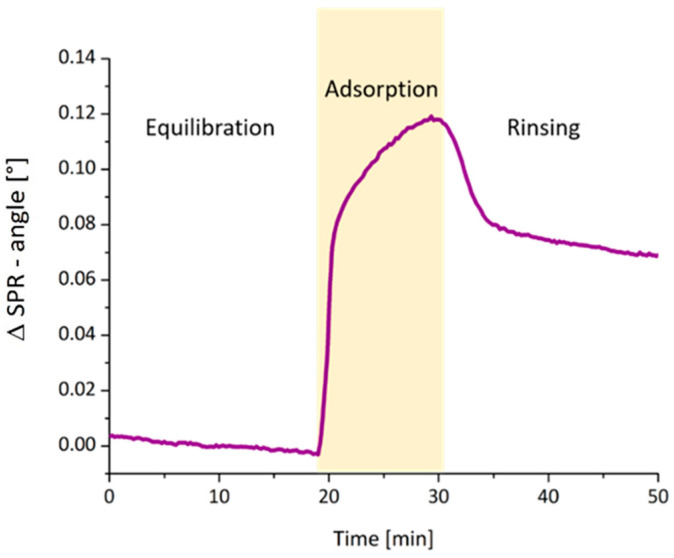
SPR-Sensogram showing the adsorption of GM (c = 1.0 mg·mL^−1^, flow rate 20 μL·min^−1^) onto cellulose surfaces measured by MP-SPR with λ = 785 nm using PBS as running buffer.

After preparation of the cellulose film, the next step was to render these surfaces with GM using an in-situ adsorption experiment using SPR. This adsorption experiment was designed in a way that after equilibration of the cellulose thin film in PBS buffer, the GM was pumped over the cellulose surface for a period of at least 10 min. The GM adsorbs onto the cellulose thin film (Figure 1). Afterwards, the film was rinsed with PBS buffer, thereby removing loosely bound material. By employing the De Fejter equation, the amount of deposited material was determined (0.64 ± 0.22 mg·m^−2^, averaged from 12 runs). 

**Figure 2:**
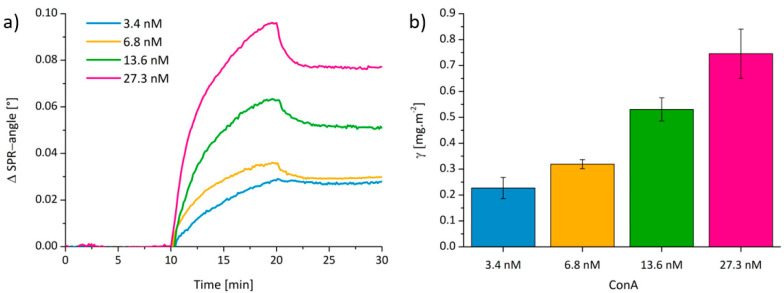
(**a**) SPR sensograms showing the adsorption of ConA at different concentrations on cellulose surfaces with pre-adsorbed GM (injection at t = 10 min, rinsing at t = 20 min) and (**b**) corresponding calculated protein adsorption after rinsing with buffer. Measurements were performed in triplicates at λ = 785 nm using PBS as buffer (flow rate: 20 µL∙min^−1^).

The next step was to explore the interaction of the ConA with the GM surfaces. We employed different concentrations of ConA to determine the binding constants with the GM rendered cellulose surfaces (Figure 2a). The adsorbed amounts of ConA (Figure 2b) rise almost linearly with concentration from Γ = 0.23 ± 0.04 (3.4 nM), 0.32 ± 0.02 (6.8 nM), 0.53 ± 0.05 (13.6 nM) to 0.75 ± 0.10 mg·m^−2^ for the highest concentration of 27.3 nM ConA. The corresponding dissociation constant KD resulting from these experiments is 2.1 ± 0.8 × 10^−8^ M.

**Figure 3:**
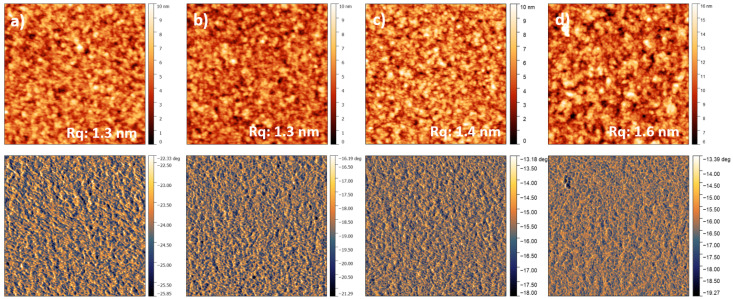
Representative 2 × 2 μm^2^ AFM topography (upper low) and phase (lower row) images of (**a**) 3.4, (**b**) 6.8, (**c**) 13.6 and (**d**) 27.3 nM ConA adsorbed from PBS-buffer on regenerated TMSC thin films with galactomannan adsorbed recorded in air at ambient conditions.

The AFM images (Figure 3) do not reveal large differences in morphology between the different samples on first glance. There are, however, subtle variations between the different ConA concentrations. Particularly, the surfaces with the highest amount of ConA used for the adsorption look more covered with the lectin compared to those with lower ConA amounts. 

The main adsorption mechanism of the lectins on the galactomannan rendered surfaces is not clear from these studies. The first thought was to check whether there is a correlation between the amount of adsorbed GM and the extent of irreversible protein deposition. There were slight deviations in the adsorption of the GM on the cellulose surface, probably due to slight variations in roughness of the cellulose film, but we could not observe any trend that more GM on the films led to more deposited protein nor the other way around. In all cases, the difference between the individual experiments was rather low. The second idea was to investigate which type of specific interactions could come into play. 

ConA is a lectin which has a high binding affinity to terminal and *α*-linked-D-mannose and, to a lesser extent, to *α*-D-glucose [39]. Previous investigations based on allowed orientations of the ConA binding site showed that the terminal mannose residues have higher probability of reaching and form more stable complexes than internal mannose residues [40,41]. If the oligosaccharide fragments contained the same terminal monosaccharide and differs in the type of linkage it may align in a different way with respect to the protein, leading to favorable or unfavorable interactions that would limit the number of carbohydrate groups to which ConA can bind. The restrictions on the oligosaccharide fragments will be much more stringent if ConA has to bind to an internal mannose residue [42], and therefore ConA binds more effectively to terminal D-mannose residues.

One option is that in this experiment the mannose reducing end of the GM interacts with one of the active sites of ConA, thereby forming specific interactions. However, in the GM under investigation here, the glycosidic linkages of the mannose residues are in a *β*-anomeric configuration. The other option would be that the ConA interacts with glucose moieties of the cellulose support. However, we found that ConA adsorption on cellulose is negligible (0.11 mg·m^−2^) and hardly reaches even the extent of BSA (bovine serum albumin) adsorption (0.22 mg·m^−2^), a commonly used marker for non-specific protein adsorption, on cellulose surfaces under the experimental conditions (PBS, pH 7.4, c = 13.6 nM). 

Most interaction studies reported with lectins have been performed for mono- and oligosaccharides (very often in solution) and here only the most affine ones have been explored. However, it is clear that also carbohydrates with a lower affinity will bind to the lectins which may be stabilized in the case of polysaccharides by additional interactions [42]. For instance, galectin-3 selectively binds to *β*-galactosyl residues in its canonical binding site [43,44] but also binds with lower affinity with α-galactosyl residues of galactomannans in the noncanonical carbohydrate-binding site on the F-face of the Gal-3 CRD β-sandwich, and to a less extent, if at all, with the canonical carbohydrate-binding site on the S-face [41]. In the case of the lectin ConA, Goldstein et al. suggested already that it binds to the terminal D-mannose unit at the reducing end of the carbohydrate chain [41]. In the hemicellulose GM, there are a variety of side chains as well as the mannose reducing end available for this type of interactions. Although the specificity for these carbohydrate moieties might be quite small, cooperative effects may come into play. This involves a reduced solubility at the GM-lectin interface which may lead to association effects which are entropically driven [45,46,47]. Further, hydrophobic effects may play a role (e.g., via van der Waals interactions) since the GM is a non-ionic polysaccharide carrying hardly any charges. However, these effects may contribute, but cannot be the driving force of and probably do not completely control the interaction between GM and the lectins. Otherwise, BSA would interact stronger with the GM rendered surfaces, since it adsorbs spontaneously on nearly all surfaces. Therefore, one of the explanations for the interaction is that the sugar moieties of the GM interact with ConA in a very weak fashion, but the reduced solubility at the interface in combination with hydrophobic interactions lead to spontaneous assembly and stabilization on the GM rendered cellulose surfaces.

The value for the dissociation constant that we obtained (2.1 ± 0.8 × 10^−8^ M) is lower as in one of the few reported mannan-lectin studies (2.9 × 10^−6^ M), where QCM-D was used to study the interaction of the mannan in solution with immobilized ConA on a solid substrate [21]. The differences in K_D_ may have several origins, including variations in the mannan type (i), immobilization method and experimental setup (ii) and the use of different techniques (iii). The influence of the mannan type can be easily rationalized as different side chains may offer a different binding site profile, leading to variations in the dissociation constant. Another important parameter is molar mass, which may lead to steric hindrance and restricted access to binding sites. The immobilization method itself can have an impact as usually the lectins are immobilized in lectin-carbohydrate assays on the surface as they are rather large molecules compared to the analytes such as mono-, oligo and, in some cases, polysaccharides. The disadvantage of immobilization can be partial denaturation of the lectin, concomitant with a loss of active sites. The advantage of the protein immobilization method includes the absence of diffusion limitation, at least when analytes are small in size such as mono and oligosaccharides. As our GM features a rather high molar mass (1 × 10^6^ g·mol^−1^), there will be some diffusion limitation and this could be one reason why the dissociation constant is two magnitudes smaller than for the reported literature value where molar mass of the mannan (obtained from *Saccharomyces cerevisiae*) was reported 54 kDa. Different methods may also yield different results on the deposited amounts. For instance, QCM-D is a gravimetric method which requires to address bound water in biomolecules layers by modelling (e.g., Voight modelling) if the Sauerbrey condition (rigid layer, no dissipation) is not fulfilled. In contrast, SPR measures the optical properties of a layer and does not consider a priori the bound water in such layers. However, also here there are approximations required, particularly if, e.g., dn/dc values are not at hand. 

## 4. Conclusions

The adsorption of the GM was studied in situ by MP-SPR spectroscopy and revealed irreversible adsorption on the cellulose thin films. Onto these surfaces, ConA was adsorbed at different concentrations and the dissociation constant K_D_ was determined. Although K_D_ is small, the deposited amounts of ConA onto the surface significantly exceeds the deposition on neat cellulose surfaces, which have been successfully studied as protein repellant surfaces in earlier reports. Furthermore, BSA adsorption on GM rendered cellulose surfaces was much lower than the ConA deposition. The mechanism is not completely clear, but the facts point at weak affinity of the carbohydrate moieties of the GM (i.e., mannose reducing end and galactose side chains) to the ConA, which cooperatively act with hydrophobic interactions (e.g., van der Waals) and entropic contributions [48]. 

It is well known that lectins are involved in many physiological processes whereas their action is often modulated by specific saccharide interactions. A better understanding of the interaction of lectins with mono-, oligo- and polysaccharides may provide a means to develop novel and more efficient drugs for a variety of diseases. Insights from this study can be used in further developments of affinity assays of different polymeric galactomannans with a variety of lectins. In particular, GMs on cellulose provide a system, whose manufacturing in the laboratory is facile and reproducible. The GM rendered surfaces can be screened for a variety of lectins to create a library of binding affinities, which can be relevant for those lectins that are involved in pathological processes.
